# Nonstructural Protein 1 of Influenza A Virus Interacts with Human Guanylate-Binding Protein 1 to Antagonize Antiviral Activity

**DOI:** 10.1371/journal.pone.0055920

**Published:** 2013-02-06

**Authors:** Zixiang Zhu, Zixue Shi, Wenjun Yan, Jianchao Wei, Donghua Shao, Xufang Deng, Shaohui Wang, Beibei Li, Guangzhi Tong, Zhiyong Ma

**Affiliations:** 1 Department of Veterinary Public Health, Shanghai Veterinary Research Institute, Chinese Academy of Agricultural Sciences, Shanghai, China; 2 Department of Swine Infectious Disease, Shanghai Veterinary Research Institute, Chinese Academy of Agricultural Sciences, Shanghai, China; University of California, Riverside, United States of America

## Abstract

Human guanylate-binding protein 1 (hGBP1) is an interferon-inducible protein involved in the host immune response against viral infection. In response to infection by influenza A virus (IAV), hGBP1 transcript and protein were significantly upregulated. Overexpression of hGBP1 inhibited IAV replication in a dose-dependent manner *in vitro*. The lysine residue at position 51 (K51) of hGBP1 was essential for inhibition of IAV replication. Mutation of K51 resulted in an hGBP1 that was unable to inhibit IAV replication. The viral nonstructural protein 1 (NS1) was found to interact directly with hGBP1. K51 of hGBP1 and a region between residues 123 and 144 in NS1 were demonstrated to be essential for the interaction between NS1 and hGBP1. Binding of NS1 to hGBP1 resulted in a significant reduction in both GTPase activity and the anti-IAV activity of hGBP1. These findings indicated that hGBP1 contributed to the host immune response against IAV replication and that hGBP1-mediated antiviral activity was antagonized by NS1 via binding to hGBP1.

## Introduction

Influenza A virus (IAV) is single-stranded negative-sense segmented RNA virus that causes influenza in humans and many animal species [1], [2]. The IAV genome consists of 8 RNA segments that encode at least 11 known proteins such as hemagglutinin (HA), neuraminidase (NA), matrix protein 1 (M1), and nonstructural protein 1 (NS1). NS1 is encoded by viral segment 8 and has multiple accessory functions during viral infection [3]. NS1 is an approximately 26 kDa protein comprising 228–237 amino acids depending on the viral strain. It is structurally divided into two distinct functional domains: an N-terminal RNA-binding domain (residues 1–73) and a C-terminal effector domain (residues 74–230). The C-terminal effector domain predominantly mediates interactions with a number of host cell proteins by which IAV antagonizes the host immune response, especially the limitation of interferon (IFN) production and the antiviral effects of IFN-induced proteins [3]. For instance, NS1 interacts with retinoic acid-inducible gene I (RIG-I) and inhibits RIG-I-mediated induction of IFN-β [4]. NS1 binds to dsRNA-activated antiviral protein kinase (PKR) to sequester the antiviral ability of PKR [5], [6].

Viral infection activates the IFN signaling pathway and induces the expression of numerous IFN-induced proteins, most of which defend cells from viral infection [7]. The IFN-induced proteins include the guanosine 5′- triphosphatase (GTPase) family, which is induced by the IFN response in a variety of organisms. The GTPase family is largely divided into four subfamilies: the Mx proteins, the very large inducible GTPases, the p47 immunity-related GTPases (IRGs), and the p65 guanylate-binding proteins (GBPs) [8]. These four GTPase subfamilies share several properties such as being regulated by IFN and being involved in the immune response against viral or microbial infections [9]. Mx proteins inhibit the replication of a wide range of viruses [10]. IRGs are reported to be essential for resisting bacterial and protozoal infection [11], [12]. GBPs are necessary for host mediation of the immune response after the invasion of many exogenous pathogens, and for resistance against bacteria, toxoplasma, chlamydia and various viruses [13]–[17].

GBPs are IFN-induced proteins with a relative molecular mass of 67–69 kDa. They contain an N-terminal globular GTPase domain and a C-terminal α-helical regulatory domain that are connected by a short middle domain [18]. GBPs bind GTP and hydrolyze it to GDP and subsequently to GMP [19]. In humans, seven GBPs (hGBP1 to hGBP7) have been identified that have a high degree of sequence homology [20]. The hGBP1 is the most well-characterized member of the GBPs. It is inducible by type I and II IFNs and contributes to the host immune response [20], [21]. The hGBP1 has been found to mediate antiviral effects against vesicular stomatitis virus (VSV), encephalomyocarditis virus (EMCV), coxsackie virus, hepatitis B virus and hepatitis C virus (HCV) [17], [22], [23]. In response to IAV infection, porcine GBP1 is upregulated at the transcriptional level in the lungs of IAV-infected pig [24]. We also observed that murine GBP1 was significantly upregulated in the lungs of IAV-infected mice (unpublished data). The upregulation of hGBP1 by IFN-γ treatment is dramatically attenuated in IAV-infected cells compared with uninfected cells [25]. These observations imply that GBP1 has potential antiviral activity against IAV infection.

The potential antiviral activity of hGBP1 against IAV infection was mentioned briefly, with limited data, in a study that described the antiviral activity of a splice variant of hGBP3 [26]. In this study, we analyzed the anti-IAV effect of hGBP1 in detail and explored the mechanism by which IAV antagonizes hGBP1-mediated antiviral activity. We found that the hGBP1 possessed antiviral activity against IAV replication and that this activity was antagonized by NS1 via binding to hGBP1.

## Materials and Methods

### Viruses, cells, infection and antibodies

Influenza viruses A/Puerto-Rico/8/34 (H1N1 subtype; PR8), A/Swine/Jiangsu/2/2006 (H3N2 subtype) and A/Swine/Gangxi/7/07 (H9N2 subtype) were propagated in the allantoic cavities of nine-day-old embryonated specific-pathogen-free chicken eggs or in Madin–Darby canine kidney cells (MDCK (NBL-2)). Human lung epithelial A549 cells (A549) were maintained in F-12K Nutrient Mixture, Kaighn's Modification (Invitrogen, Carlsbad, CA, USA). MDCK cells and H1299 cells were maintained in Dulbecco's modified Eagle's medium (Invitrogen). MDCK (NBL-2), A549 and H1299 cells were purchased from the Cell Bank of Type Culture Collection of Chinese Academy of Sciences (Shanghai, China). All cells were cultured in their medium supplemented with 10% fetal bovine serum at 37°C under 5% CO_2_. Viral infection was as described previously [27]. All animal experiments were performed in compliance with the Guidelines on the Humane Treatment of Laboratory Animals (Ministry of Science and Technology of the People's Republic of China, Policy No. 2006 398) and were approved by the Institutional Animal Care and Use Committee at the Shanghai Veterinary Research Institute, Chinese Academy of Agricultural Science. Commercial antibodies were anti-Flag monoclonal antibody (M2, Sigma, St. Louis, MO, USA), anti-hGBP1 monoclonal antibody (1B1, Santa Cruz Biotechnology, Santa Cruz, CA, USA), anti-Myc monoclonal antibody (sc-40, Santa Cruz Biotechnology), and anti-β-actin monoclonal antibody (AC-15, Sigma). Anti-NP polyclonal antibody and anti-NS1 polyclonal antibody were generated in our laboratory (unpublished data).

### Plasmids and transfection

A plasmid expressing Myc-tagged wild-type hGBP1 (Myc-hGBP1-wt) was constructed by inserting full-length hGBP1 cDNA into pcDNATM3.1/myc-His A vector (Invitrogen). A plasmid expressing Flag-tagged wild-type NS1 (Flag-NS1-wt) was constructed by inserting full-length NS1 cDNA of PR8 strain into p3xFLAG-CMV-7.1 vector (Sigma). A series of plasmids expressing Flag-tagged truncated mutants of NS1 and Myc-tagged hGBP1 mutant (Myc-hGBP1-K51A) were generated by modified PCR-based site-directed mutagenesis. For bimolecular fluorescence complementation (BiFC) assays, sequences encoding the N- (amino acids 1–173, VN) and C- (amino acids 174–239, VC) terminal fragments of venus fluorescent protein [28] were fused by a short linker to hGBP1 (hGBP1-VN) and NS1 gene (NS1-VC), respectively [29]. An hGBP1 mutant fused to VN (hGBP1-K51A-VN) was obtained by modified PCR-based site-directed mutagenesis. β-actin was fused to VN (actin-VN) and VC (actin-VC) in the same way as controls. All recombinant plasmids were verified by DNA sequencing. Cells were transfected using Lipofectamine™ 2000 (Invitrogen), according to the manufacturer's protocol.

### Quantitative real-time RT-PCR (qRT-PCR)

Total RNA was extracted from cells using the TRIzol® Reagent (Invitrogen) according to the manufacturer's protocol. Two micrograms of total RNA were used to synthesize cDNA using M-MLV reverse transcriptase (Invitrogen). The qRT-PCR for analysis of viral gene expression was performed using SYBR Premix Ex Taq™ (Takara, Kyoto, Japan) according to the manufacturer's protocol. The housekeeping gene glyceraldehyde-3-phosphate dehydrogenase (GAPDH) was used as an internal control. Primer sequences are in Table S1.

### Plaque assay

MDCK cell monolayers grown in 6-well culture plates were incubated with serial dilutions of viral inoculum at 37°C for 1 h. Plates were shaken gently every 20 min during incubation to ensure well-distributed viral adsorption. After 1 h, inoculum was replaced with 1% LMP agarose (invitrogen) in UltraMDCK Serum-Free medium (Lonza, Walkersville, MD, USA) with 1.25 µg/ml TPCK trypsin. Following agarose solidification, plates were incubated at 37°C with 5% CO_2_ for 3 days. Plaques were counted manually and the number of plaque forming units per ml was calculated.

### Indirect immunofluorescence assay

A549 cells grown on cover slips were fixed in methanol and acetone mixture (1∶1) at −20°C for 10 min. Cells were blocked with 10% goat serum in phosphate buffer saline at 37°C for 30 min and probed with anti-NS1 and anti-Myc primary antibodies at 37°C for 30 min. Antibody-antigen complexes were detected with Alexa Fluor**®** 488 conjugated anti-rat IgG and Alexa Fluor**®** 546 conjugated anti-mouse IgG secondary antibodies (Invitrogen) by incubation for 30 min at 37°C. Nuclei were counterstained with 4′,6′-diamidino-2-phenylindole (DAPI). Stained cells were visualized using a fluorescence microscope.

### Immunoprecipitation and western blot analysis

H1299 cells grown in 100 mm plates were transiently co-transfected with 5 µg of Myc-hGBP1-wt and 5 µg of Flag-NS1-wt or Flag-NS1-mutants constructs. The transfectants were harvested 36 h after transfection and subjected to immunoprecipitation assay. The cells were lysed in NP-40 lysis buffer (1% NP-40, 50 mM Tris (pH 8.0), 5 mM EDTA, 150 mM NaCl, 2 mg/ml leupeptin, 2 mg/ml aprotinin, 1 mM phenylmethanesulfonyl fluoride) and pre-cleared by incubation with protein G agarose beads (Sigma) for 1 h at 4°C. The lysates were then incubated with anit-Myc or anti-Flag antibodies on a rotating wheel overnight at 4°C. Protein G agarose beads were then added and the mixtures were further incubated on a rotating wheel for 2 h at 4°C. The agarose beads were pelleted and washed three times in NP-40 lysis buffer. Antibody–antigen complexes bound to the beads were eluted in SDS-PAGE sample buffer by boiling, resolved by SDS-PAGE, and analyzed by western blot analysis with the appropriate antibodies.

For western blot, the cells were lysed in a lysis buffer (1% SDS, 1% NP-40, 50 mM Tris (pH8.0), 150 mM NaCl, 4 mM Pefabloc SC, 2 mg/ml leupeptin, 2 mg/ml aprotinin). The lysates were briefly sonicated, boiled for 5 min and then centrifugated for 10 min at 20, 000 g at 4°C. The concentration of protein in the lysates was assessed with the BCA protein assay reagent (Pierce Biotechnology, Rockford, IL, USA). The lysates were further denatured by a 5 min incubation in a sample buffer (2% SDS, 10% glycerol, 60 mM Tris (pH 6.8), 5% β-mercaptoethanol and 0.01% bromophenol blue) at 95°C. The samples were then subjected to SDS-PAGE, and transferred to Immobilon-P membranes (Millipore Corp., Bedford, MA, USA), which were incubated first in blocking buffer (5% non-fat milk powder in Tris-buffered saline containing 0.2% Tween 20 (TBS-T)) for 1 h at room temperature, and subsequently in a solution of primary antibody for 16 h at 4°C. Finally, the membranes were washed in TBS-T to remove unbound primary antibody, and then incubated with horseradish peroxidase-conjugated secondary antibody for 1 h at room temperature. After a further wash in TBS-T, the antigen-antibody complex was visualized by enhanced chemiluminescence (Amersham Pharmacia Biotech, Piscataway, NJ, USA). The change in abundance of NP in the transfectants was determined by densitometric analysis. The ratio between NP and β-actin was plotted.

### Bimolecular fluorescence complementation (BiFC) assay

A549 cells grown in 35 mm plates were transiently co-transfected with combinations of plasmids hGBP1-VN (1 µg) and NS1-VC (1 µg), hGBP1-VN (1 µg) and actin-VC (1 µg), actin-VN (1 µg) and NS1-VC (1 µg), or VN-vector (1 µg) and VC-vector (1 µg) and incubated at 37°C for 20 h. Fluorescence emission was detected using a fluorescence microscope.

### Guanosine triphosphatase (GTPase) assay

A549 cells were transiently co-transfected with combinations of plasmids Myc-hGBP1-wt and Flag-NS1-wt, Myc-hGBP1-wt and Flag-NS1-Δ123/144 (a truncated mutant), Myc-hGBP1-wt and Flag-vector, Myc-hGBP1-K51A and Flag-vector, or Myc-vector and Flag-vector and incubated for 36 h. GTPase activity in transfectants was determined using an ATPase/GTPase ELIPA Biochem Kit (Cytoskeleton, Denver, CO, USA) that measures the amount of inorganic phosphate (Pi) generated during hydrolysis on a real-time basis, according to the manufacturer's instructions.

### Statistical analysis

All measured values are expressed as the means with standard deviations (SD). Significance was analyzed using the Student's *t*-test. Values of *p*<0.05 were considered significant.

## Results

### Dynamics of hGBP1 expression in response to IAV infection

**Figure 1:**
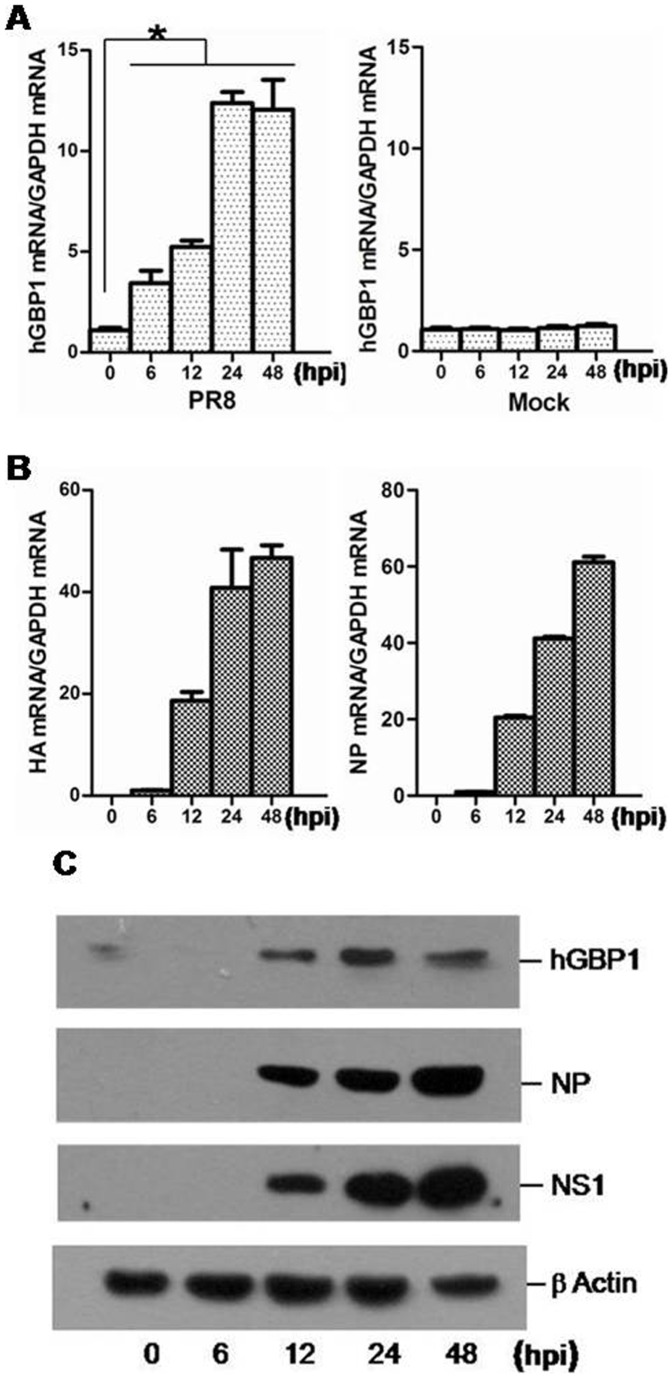
Upregulated expression of hGBP1 in PR8-infected cells. A549 cells were infected with PR8 strain or mock-infected (Mock) and collected at 0, 6, 12, 24 and 48 h post-infection (hpi). ***A.*** The hGBP1 mRNA was detected by qRT-PCR. ***B.*** Viral HA and NP mRNA in PR8-infected A549 cells was detected by qRT-PCR. ***C.*** The hGBP1, viral NS1 and NP in PR8-infected A549 cells was detected by western blot. Results are means with SD from three independent experiments. *, *p*<0.05 compared with sample collected at 0 hpi.

Influenza viruses A/Puerto-Rico/8/34 (H1N1 subtype; PR8), A/Swine/Jiangsu/2/2006 (H3N2 subtype) and A/Swine/Gangxi/7/07 (H9N2 subtype) were used in this study. Because similar results were observed with all IAV strains, we show only results obtained from the PR8 strain. To analyze the dynamics of hGBP1 expression in response to IAV infection, A549 cells, which have been used for studies of IAV infection [30], were infected with PR8 virus at a multiplicity of infection (MOI) of 5 and harvested at 0, 6, 12, 24 and 48 h post-infection (hpi) for analysis of hGBP1 expression. Transcription of hGBP1 was found to be significantly upregulated starting at 6 hpi, gradually increased, and peaked at 24 hpi in PR8-infected cells. No remarkable changes in hGBP1 expression was detected in mock-infected cells (Fig. 1A). To explore the correlation between hGBP1 expression and virus replication, we examined viral hemagglutinin (HA) and nucleoprotein (NP) expression. Expression of HA and NP was undetectable until 6 hpi (when hGBP1 was significantly upregulated), and gradually increased, similar to hGBP1 (Fig. 1B). We also detected hGBP1, NP and NS1 protein using western blots. As shown in Figure 1C, a remarkable increase at hGBP1 protein level was detected in PR8-infected cells. These results showed that the expression of hGBP1 was significantly upregulated in response to IAV infection.

### Overexpression of hGBP1 inhibits IAV replication

**Figure 2:**
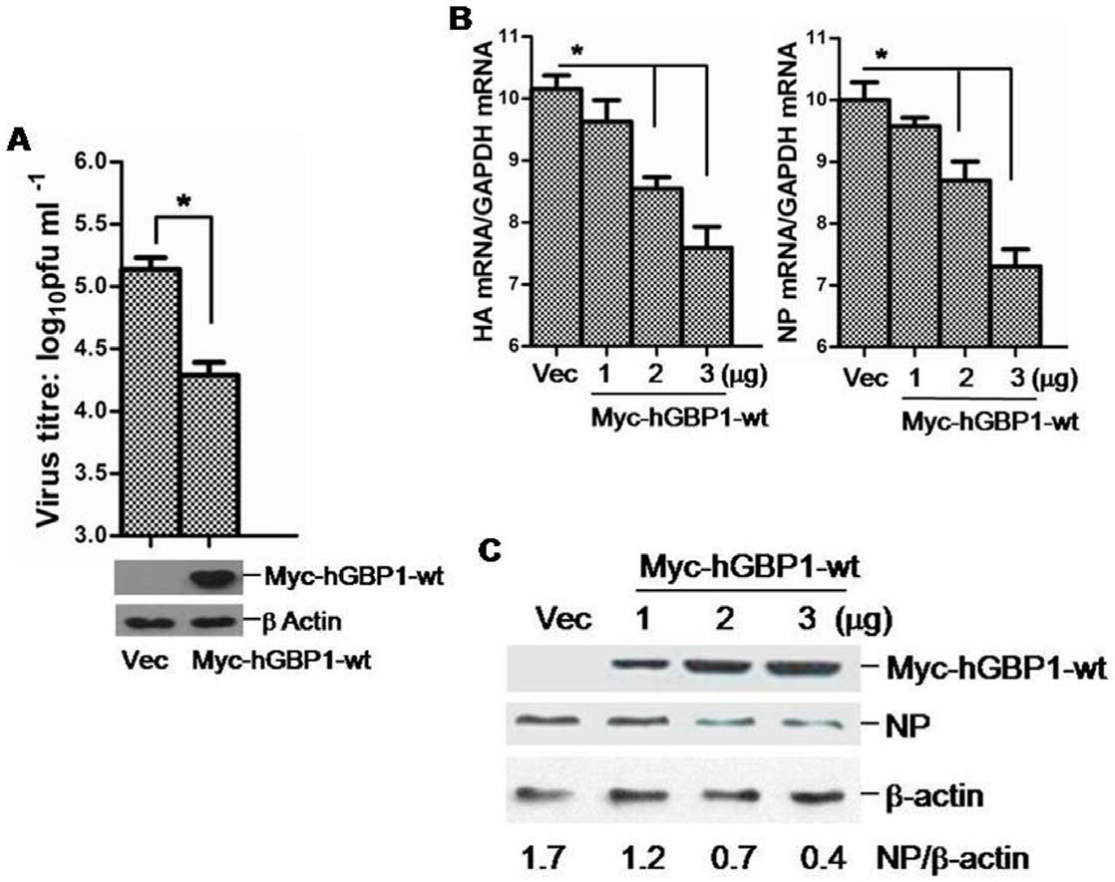
Overexpression of hGBP1 inhibited PR8 replication. ***A.*** A549 cells were transfected with plasmid Myc-hGBP1-wt or empty vector (Vec) and infected with PR8 at MOI = 1 after 24 h. Viral titers in transfectants were measured by plaque assay at 24 hpi. ***B*** and ***C.*** A549 cells were transfected with increasing amounts of plasmid Myc-hGBP1-wt and infected with PR8 at MOI = 1 after 24 h. Empty vector (Vec, 3 µg) was transfected in parallel as a control. ***B.*** HA and NP mRNA in transfectants was analyzed at 24 hpi by qRT-PCR. ***C.*** Myc-hGBP1-wt and NP were detected at 24 hpi by western blot. Results are means with SD from three independent experiments. *, *p*<0.05 compared with cells transfected with empty vector.

Since hGBP1 was significantly upregulated in response to IAV infection, we transiently overexpressed hGBP1 to determine whether hGBP1 had an inhibitory effect on IAV replication. A549 cells were transfected with a plasmid expressing Myc-hGBP1-wt and subsequently infected with PR8 virus after 24 h. Cells were harvested at 24 hpi for analysis of viral replication. Expression of Myc-hGBP1-wt was confirmed by western blot (Fig. 2A). Plaque assays indicated that viral titers were significantly reduced in Myc-hGBP1-wt-transfected cells compared with mock-transfected cells (Fig. 2A). To determine whether the inhibitory effect of hGBP1 on IAV replication was dose dependent, A549 cells were transfected with increasing amounts of Myc-hGBP1-wt and subsequently infected with PR8 virus. The relative abundance of HA and NP mRNA was used as an indicator of viral replication. As shown in Figure 2B, HA and NP mRNA gradually decreased with increases in Myc-hGBP1-wt expression, showing that hGBP1 acted in a dose-dependent manner to inhibit IAV replication. This observation was further confirmed by western blot for NP in Myc-hGBP1-wt-transfected cells (Fig. 2C). These results suggested that hGBP1 inhibited IAV replication.

### Lysine at position 51 (K51) of hGBP1 is required for inhibition of IAV replication

**Figure 3:**
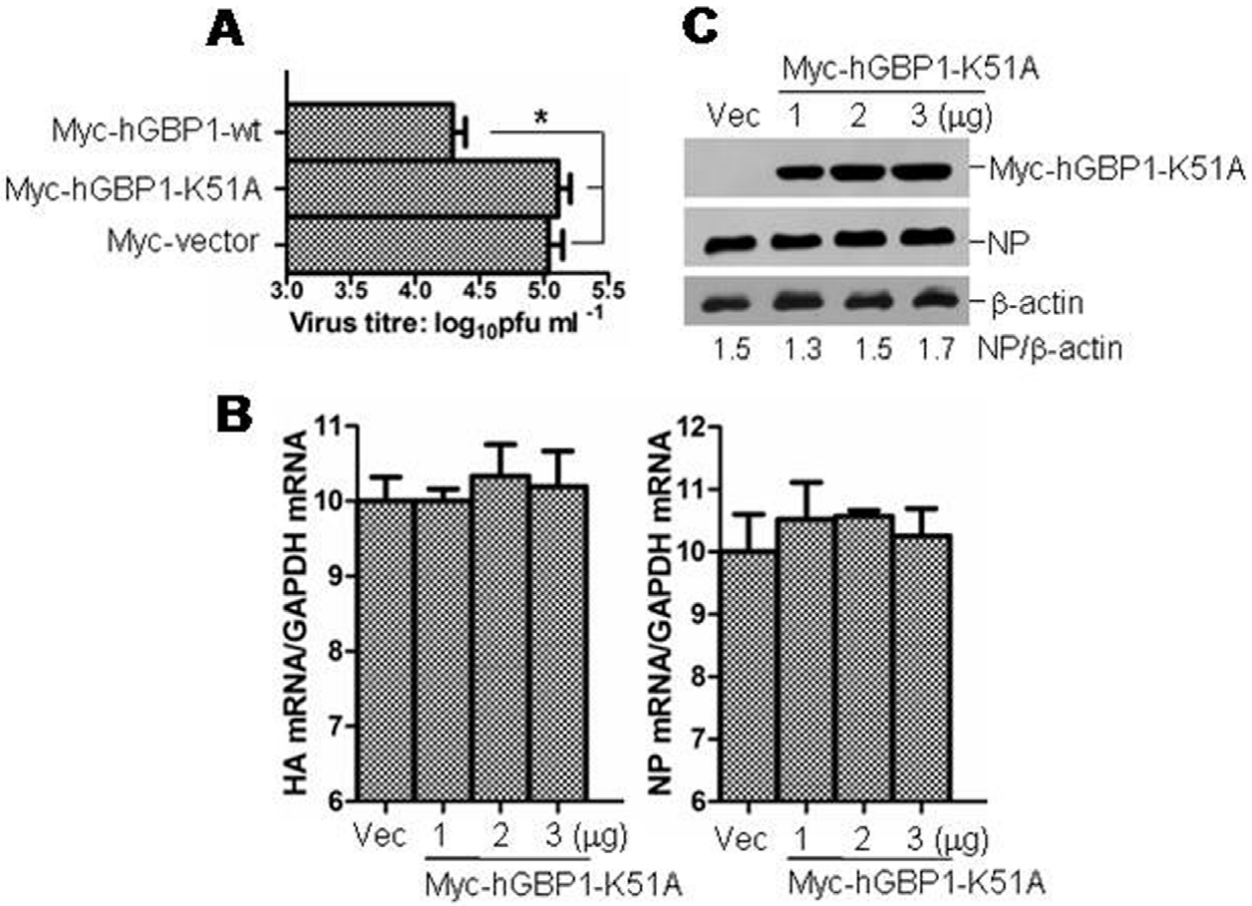
Overexpression of hGBP1-K51A did not inhibit IAV replication. ***A.*** A549 cells were transfected with plasmid Myc-hGBP1-wt, Myc-hGBP1-K51A or Myc-vector and infected with PR8 at MOI = 1 after 24 h. Viral titers in transfectants were measured by plaque assay at 24 hpi. ***B*** and ***C.*** A549 cells were transfected with increasing amounts of plasmid Myc-hGBP1-K51A and infected with PR8 at MOI = 1 after 24 h. Empty vector (Vec, 3 µg) was transfected in parallel as a control. ***B.*** HA and NP mRNA in transfectants was analyzed at 24 hpi by qRT-PCR. ***C.*** Myc-hGBP1-K51A and NP were detected at 24 hpi by western blot. Results are means with SD from three independent experiments. *, *p*<0.05 compared with cells transfected with Myc-hGBP1-wt.

The K51 located in the highly conserved phosphate-binding loop of hGBP1 is critical for its biological activity and function. Mutation of lysine 51 to alanine (K51A) abrogates GTP binding, dimerization, and GTP hydrolysis [31]. To test whether K51 of hGBP1 is essential for inhibition of IAV replication, A549 cells were transiently transfected Myc-hGBP1-wt or Myc-hGBP1-K51A and subsequently infected with PR8 virus. Expression of Myc-hGBP1-wt significantly reduced the viral titer compared with mock-transfected cells, consistent our other observations (Fig. 2A). However, expression of Myc-hGBP1-K51A showed no remarkable change in viral titer compared with mock-transfected cells; however, cells with this plasmid had a viral titer significantly higher than cells with Myc-hGBP1-wt (Fig. 3A). We also detected HA and NP mRNA in Myc-hGBP1-K51A-transfected and mock-transfected cells. No detectable difference in mRNA was observed of either viral genes in Myc-hGBP1-K51A- and mock-transfected cells (Fig. 3B). NP expression was also compared between Myc-hGBP1-K51A-transfected and mock-transfected cells by western blot. In contrast to the observation that overexpression of Myc-hGBP1-wt reduced NP (Fig. 2C), overexpression of Myc-hGBP1-K51A did not reduce NP compared with mock-transfected cells (Fig. 3C). These data indicated that K51 of hGBP1 was required for inhibition of IAV replication.

### Viral NS1 interacts with hGBP1

**Figure 4:**
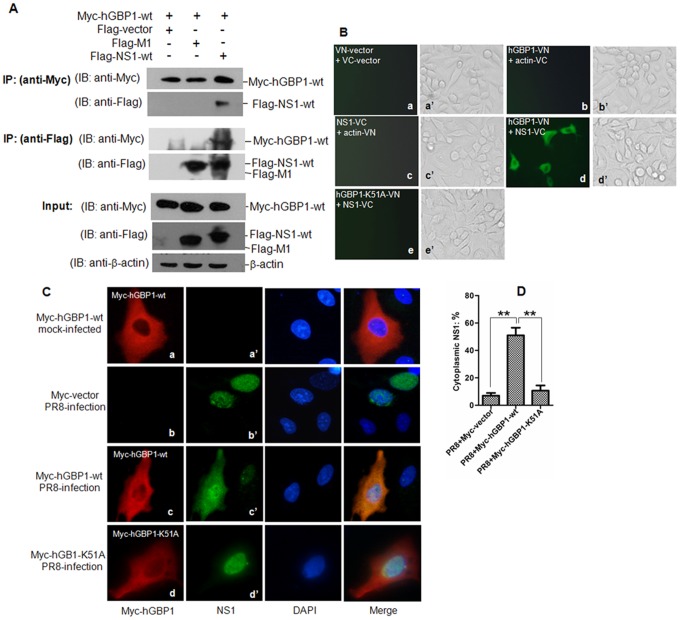
Detection of interaction between NS1 and hGBP1. ***A.*** Immunoprecipitation assay for detecting interaction between NS1 and hGBP1. H1299 cells were transfected with the indicated plasmids. Transfectants were harvested after 36 h and subjected to immunoprecipitation and western blot with anti-Myc or anti-Flag. IP, immunoprecipitation. IB, western blot. ***B.*** BiFC assay for detecting interaction between NS1 and hGBP1. A549 cells were transiently transfected with indicated plasmids and incubated for 20 h. Fluorescence emission and brightfield were visualized. ***C.*** Indirect immunofluorescence assay for detecting colocalization of NS1 and hGBP1. A549 cells were transfected with plasmid Myc-hGBP1-wt or Myc-vector and incubated for 12 h before infection with PR8 at MOI = 1 or mock-infection and incubation for 24 h. Cells were double-immunostained for Myc-hGBP1 (red) and NS1 (green). Nuclei were counterstained with DAPI (blue). ***D.*** Percentage of cells with cytoplasmic localization of NS1. The number of cells with cytoplasmic localization of NS1 in ten randomly selected visual fields was counted. The percentage of cells with cytoplasmic localization of NS1 was calculated and plotted. Results are presented as the means ± S.E. from three independent experiments. **, *p*<0.01 compared with cells transfected with PR8+Myc-hGBP1-wt.

Viral NS1 interacts with cellular proteins including IFN-induced proteins to antagonize the host immune response against viral infection [3]. We therefore hypothesized that NS1 interacted with hGBP1 and inhibited its antiviral activity. To detect whether NS1 interacted with hGBP1, we transiently co-expressed Myc-hGBP1-wt with Flag-tagged wild type NS1 (Flag-NS1-wt) in H1299 cells. Transfectants were subjected to immunoprecipitation assays with anti-Myc or anti-Flag antibodies. Flag-tagged viral matrix protein 1 (Flag-M1) was co-expressed in parallel with Myc-hGBP1-wt as a control. Protein in transfectants was determined by western blot (Fig. 4A, input panels). Myc-hGBP1-wt immunoprecipitated Flag-NS1-wt, but did not pull down Flag-M1 (Fig. 4A, anti-Myc panels). A reverse immunoprecipitation assay was performed using anti-Flag antibody. Flag-NS1-wt, but not Flag-M1, immunoprecipitated Myc-hGBP1-wt (Fig. 4A, anti-Flag panels).

To detect whether NS1 directly interacted with hGBP1, a BiFC assay, which detects direct protein-protein interactions in living cells [32], [33], was performed (Fig. S2). A549 cells were transiently transfected with combinations of indicated plasmids (Fig. 4B) and fluorescence emission was assessed. No fluorescence emission was observed in cells transfected with a combination of VN-vector and VC-vector, hGBP1-VN and actin-VC, or NS1-VC and actin-VN (Fig. 4B, panel a, b, and c). However, the cells co-transfected with hGBP1-VN and NS1-VC emitted fluorescence (Fig. 4B, panel d). These results suggested direct interaction between hGBP1 and NS1.

We also detected the colocalization of NS1 and hGBP1 in PR8-infected cells. A549 cells were transiently transfected with Myc-hGBP1-wt and subsequently infected with PR8 virus. Transfectants were collected at 24 hpi for indirect immunofluorescence assays. Myc-hGBP1-wt distributed predominantly in the cytoplasm in both PR8-infected and mock-infected cells, with a diffused pattern as described previously [34] (Fig. 4C, panels a and c). Viral NS1 in PR8-infected cells showed nuclear localization with a minor cytoplasmic component (Fig. 4C, panel b′ and Fig. 4D). However, in cells transfected with Myc-hGBP1-wt, a fraction of NS1 was translocated from the nucleus to the cytoplasm (Fig. 4C, panel c′ and Fig. 4D), showing colocalization with Myc-hGBP1-wt in the cytoplasm. This cytoplasmic sequestration of NS1 by hGBP1 was also observed in cells infected with influenza A/Swine/Jiangsu/2/2006 (H3N2 subtype) (Fig. S1) and A/Swine/Gangxi/7/07 (H9N2 subtype) (data not shown). These results suggested a direct interaction between NS1 and hGBP1.

### Mapping of hGBP1-binding region of NS1

**Figure 5:**
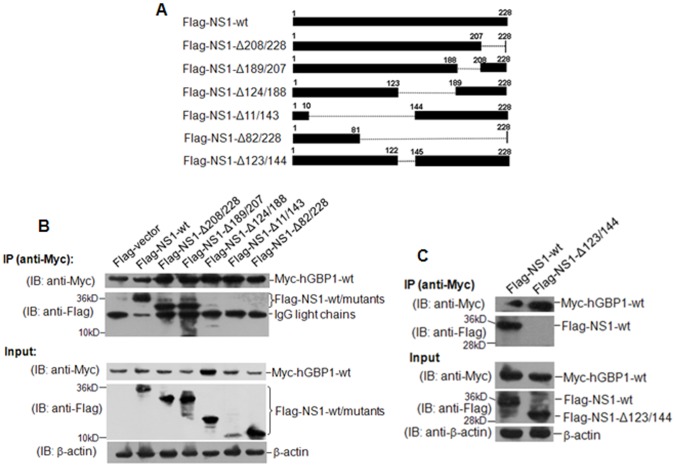
Mapping the hGBP1-binding region of NS1. ***A.*** Schematic representation of Flag-tagged wild-type NS1 and truncated mutants. ***B.*** H1299 cells were transfected with the indicated plasmids in the presence of plasmid Myc-hGBP1-wt. Transfectants were collected after 36 h and immunoprecipitated with anti-Myc antibody. ***C.*** H1299 cells were transfected with plasmid Flag-NS1-wt or Flag-NS1-Δ123/144 in the presence of plasmid Myc-hGBP1-wt. Transfectants were collected after 36 h and immunoprecipitated with anti-Myc antibody. IP, immunoprecipitation. IB, western blot.

To map the region of NS1 required for binding to hGBP1, we constructed a series of plasmids expressing Flag-tagged deletion mutants of NS1 (Fig. 5A). H1299 cells were transfected with these plasmids in the presence of Myc-hGBP1-wt and subjected to immunoprecipitation assays. Myc-hGBP1-wt immunoprecipitated.

Flag-NS1-wt, Flag-NS1-Δ208/228, and Flag-NS1-Δ189/207, but did not pull down Flag-NS1-Δ124/188, Flag-NS1-Δ11/143, or Flag-NS1-Δ82/228 (Fig. 5B). These results implied that the region from residue 123 to 144 was required for NS1 to interact with hGBP1. To verify this finding, a plasmid expressing a NS1 mutant lacking residues 123 through 144 (Flag-NS1-Δ123/144) was co-transfected with Myc-hGBP1-wt into H1299 cells for immunoprecipitation assays. As shown in Figure 5C, Flag-NS1-Δ123/144 was not immunoprecipitated by Myc-hGBP1-wt, suggesting that the region between residues 123 and 144 was essential for NS1 to bind to hGBP1.

### K51 of hGBP1 is necessary for NS1-hGBP1 interaction

**Figure 6:**
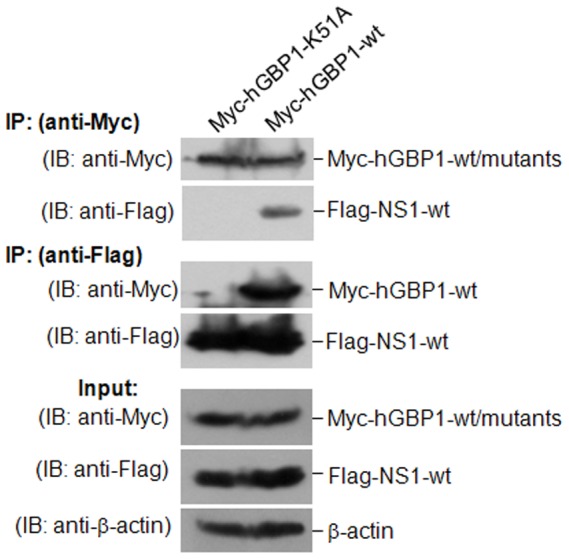
Detection of interaction between hGBP1-K51A and NS1. H1299 cells were transfected with plasmid Myc-hGBP1-wt or Myc-hGBP1-K51A in the presence of plasmid Flag-NS1-wt. Transfectants were collected after 36 h and immunoprecipitated with anti-Myc and anti-Flag antibodies. IP, immunoprecipitation. IB, western blot.

Since K51 of hGBP1 is essential for GTPase activity [31] and anti-IAV activity (Fig. 3), we determined whether K51 was required for NS1-hGBP1 interaction. A plasmid expressing Myc-hGBP1-K51A was co-transfected with Flag-NS1-wt into cells for immunoprecipitation assays. Myc-hGBP1-wt was used as control. Myc-hGBP1-wt, but not Myc-hGBP1-K51A immunoprecipitated Flag-NS1-wt (Fig. 6, anti-Myc panels). A reverse immunoprecipitation assay using anti-Flag antibody showed that Flag-NS1-wt immunoprecipitated Myc-hGBP1-wt, but did not pull down Myc-hGBP1-K51A (Fig. 6, anti-Flag panels). The requirement of K51 for NS1-hGBP1 interaction was further determined by BiFC assay. Cells co-transfected with Myc-hGBP1-K51A-VN and NS1-VC did not emit fluorescence (Fig. 4B, panel e). In addition, we detected whether Myc-hGBP1-K51A sequestered NS1 in the cytoplasm by immunofluorescence assay. In contrast to the observation that Myc-hGBP1-wt sequestrated NS1 in the cytoplasm (Fig. 4C, panel c′ and Fig. 4D), Myc-hGBP1-K51A showed no visualizable effect on the subcellular localization of NS1 (Fig. 4C, panel d′ and Fig. 4D), which was distributed predominantly in the nucleus similar to in mock-transfected cells (Fig. 4C, panel b′ and Fig. 4D). These findings indicated that the K51 was necessary for NS1-hGBP1 interaction.

### NS1 inhibits GTPase activity of hGBP1

**Figure 7:**
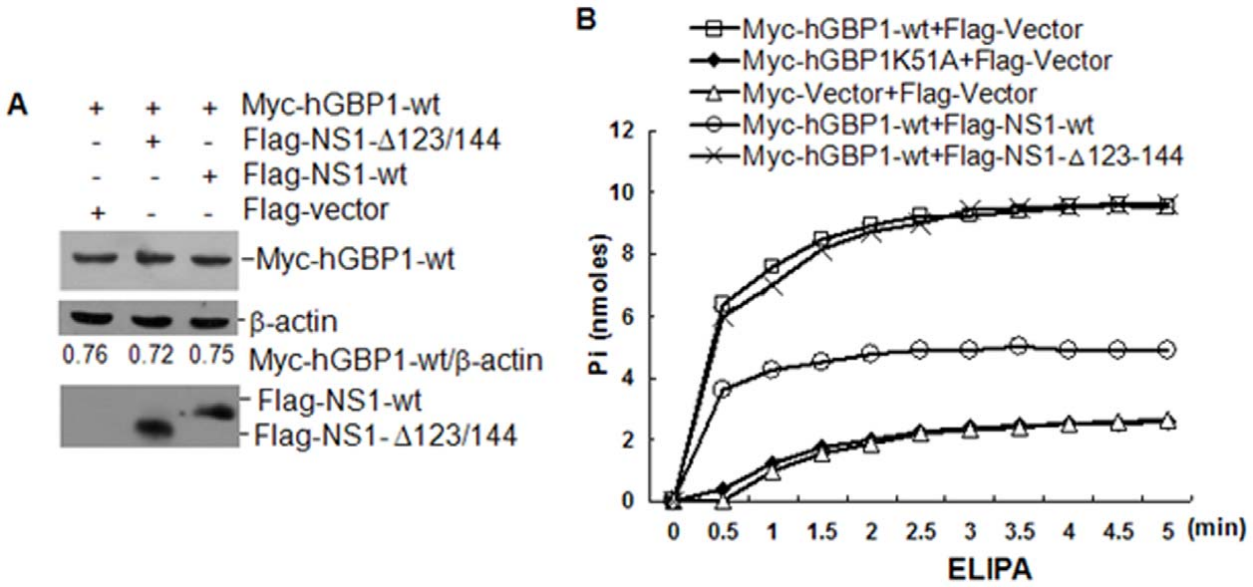
Effect of NS1 on GTPase activity of hGBP1. ***A.*** A549 cells were transfected with the indicated plasmids and collected after 36 h. Myc-hGBP1-wt, Flag-NS1-wt and Flag-NS1-Δ123/144 were detected by western blot. ***B.*** A549 cells were transfected with the indicated plasmids and collected after 36 h. GTPase activity was measured by enzyme-linked inorganic phosphate assay (ELIPA).

Because K51 of hGBP1 is essential for GTPase activity [31] and for NS1-hGBP1 interaction (Fig. 6), we hypothesized that the binding of NS1 to hGBP1 interfered with the enzymatic function of hGBP1. A549 cells were transfected with Flag-NS1-wt, Flag-NS1-Δ123/144, or Flag-vector in the presence of plasmid Myc-hGBP1-wt and cells were harvested at 36 h after transfection for western blots. No remarkable change in protein abundance of Myc-hGBP1-wt was detected between the transfectants (Fig. 7A), suggesting that Flag-NS1-wt and Flag-NS1-Δ123/144 did not interfere with Myc-hGBP1-wt expression. To analyze the effect of NS1 on the enzymatic function of hGBP1, A549 cells were transfected with combinations of plasmids Myc-hGBP1-wt and Flag-NS1-wt, Myc-hGBP1-wt and Flag-NS1-Δ123/144, Myc-hGBP1-wt and Flag-vector, Myc-hGBP1-K51A and Flag-vector, or Myc-vector and Flag-vector (Fig. 7B). Transfectants were collected after 36 h for GTPase activity analysis using enzyme-linked inorganic phosphate assays (ELIPA). GTPase activity was expressed as the amount of inorganic phosphate (Pi) released from GTP. The expression of Myc-hGBP1-wt significantly increased cellular GTPase activity, while the expression of Myc-hGBP1-K51A did not increase this activity compared with vector-transfected cells (Fig. 7B). The enhancement of GTPase activity by Myc-hGBP1-wt expression was remarkably reduced by co-expression of Flag-NS1-wt, but not by co-expression of Flag-NS1-Δ123/144 (Fig. 7B). These data suggested that NS1 inhibited the GTPase activity of hGBP1.

### NS1 antagonizes the hGBP1-mediated antiviral activity

**Figure 8:**
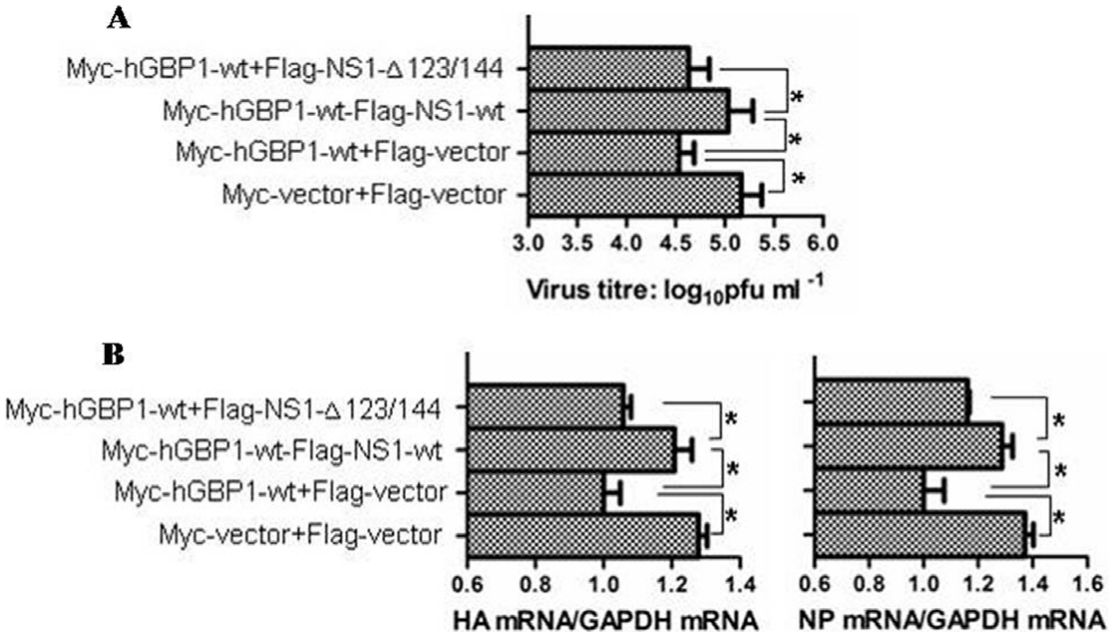
NS1 antagonized hGBP1-mediated antiviral activity. A549 cells were transfected with the indicated plasmids. Transfectants were infected with PR8 virus at MOI = 1 after 24. Cells were collected at 24 hpi for analysis of viral replication. ***A.*** Viral titers in transfectants were measured by plaque assay. ***B.*** HA and NP mRNA in transfectants was analyzed by qRT-PCR. Results are means with SD from three independent experiments. *, *p*<0.05.

NS1 is an important factor for IAV antagonism of the host immune response. NS1 interacts with several host cell proteins and consequently inhibits their antiviral activity [3]. Because NS1 interacted with hGBP1 (Fig. 4) and inhibited hGBP1 GTPase activity (Fig. 7), we determined whether NS1 interfered with the anti-IAV activity of hGBP1. A549 cells were transfected with Flag-NS1-wt, Flag-NS1-Δ123/144 or Flag-vector in the presence of Myc-hGBP1-wt and incubated for 24 h before infection with PR8 virus and incubation for 24 h. Analysis of viral titers in the transfectants indicated that expression of Myc-hGBP1-wt remarkably reduced the viral titer compared to vector-transfected cells (Fig. 8A), in agreement with other observations (Fig. 2A). This inhibitory effect of Myc-hGBP1-wt on viral replication was significantly attenuated by co-expression of Flag-NS1-wt, but not by Flag-NS1-Δ123/144, which was unable to interact with hGBP1 (Fig. 8A). These observations were confirmed by analysis of changes in HA and NP mRNA in the transfectants. Flag-NS1-wt, but not Flag-NS1-Δ123/144, significantly attenuated the inhibitory effect of Myc-hGBP1-wt on HA and NP mRNA (Fig. 8B). These findings suggested that NS1 antagonized the anti-IAV activity of hGBP1.

## Discussion

The type I IFN response protects cells against invading viral pathogens. This response is mainly mediated by a diverse range of IFN-induced proteins [7]. The GBPs are a group of IFN-induced proteins that are necessary for host immune response against pathogen infection [8]. Among 7 human GBPs, hGBP1 is the best-characterized member [20] and mediates antiviral activity against several viruses [17], [22], [23]. In addition, a wild-type and a splice variant (hGBP-3ΔC) of hGBP3 inhibit the replication of IAV and VSV [26]. The anti-IAV effect of hGBP1 has been documented with limited data in a previous study that mainly described the anti-IAV activity of hGBP-3ΔC [26]. We therefore analyzed the anti-IAV effect of hGBP1 in detail and explored a mechanism by which IAV antagonizes hGBP1-mediated antiviral activity. We found that in response to IAV infection, hGBP1 was significantly upregulated both at the transcriptional and protein level *in vitro* (Fig. 1) and that overexpression of hGBP1 significantly inhibited IAV replication in a dose-dependent manner (Fig. 2). These results were in agreement with the previous description that hGBP1 possesses an anti-IAV effect [26]. IAV is susceptible to multiple antiviral ISGs with a range of inhibitory activities [35], [36], [37], [38]. Although hGBP1 has been demonstrated to significantly inhibit IAV replication, the inhibitory effect was modest (Fig. 2). This modest antiviral effect of hGBP1 has also been described in previous studies [8]. It is known that different viruses are targeted by unique sets of ISGs, which is largely divided into two categories: i) strong inhibitors and ii) modest inhibitors [39]. Based on our observation, hGBP1 can be classified into the category of modest inhibitor. An effective IFN response requires the combinatorial action of numerous ISGs [39]. In the case of anti-IAV response, hGBP1 presumably cooperates with other ISGs to exert antiviral activity.

The biological activity and function of GBPs depend on their ability to bind and hydrolyze GTP as well as on formation of dimers and oligomers [26]. GTPase activity is important for hGBP1 to exert anti-HCV activity [17]. However, the GTPase activity of hGBP3 does not seem to be necessary for inhibition of IAV replication [26]. The GTP-binding motif of murine GBP2 is required for inhibition of EMCV replication, but not for VSV [40]. These previous observations indicated different mechanisms by which GBPs exert antiviral activity. To gain an insight into the mechanism underlying the anti-IAV effect of hGBP1, we employed a mutant (hGBP1-K51A), in which K51 was replaced with alanine. K51 is critical for hGBP1 biological activity and function including GTP-binding, dimerization, and GTPase activity [31]. Mutation of K51 resulted in a significant reduction in GTPase activity (Fig. 7). Comparison of IAV replication between cells expressing hGBP1-wt and hGBP1-K51A indicated that K51 of hGBP1 was required for inhibition of IAV replication (Fig. 3). K51 is essential for both hGBP3 and hGBP-3ΔC to inhibit IAV replication [26]. Our data, along with the previous observation [26], indicated the importance of K51 in inhibition of IAV replication. K51 is involved in GTP-binding, dimerization, and GTPase activity [31], but the exact mechanism of how the K51 contributes to the anti-IAV activity needs to be further explored. In this study, we observed that the GTPase activity of hGBP1 correlated with its anti-IAV activity. The overexpression of hGBP1 raised the cellular GTPase activity and inhibited IAV replication, while binding of NS1 to hGBP1 reduced cellular GTPase activity and attenuated the anti-IAV effect of hGBP1 (Fig. 7 and 8). These results implied that the GTPase activity of hGBP1 might be essential for inhibition of IAV replication.

The NS1 is an important factor for IAV to antagonize the host immune response and facilitate virus replication. NS1 evades IFN-mediated immune response at different steps. NS1 targets the ubiquitin ligase TRIM25 to escape from recognition by the host viral RNA sensor RIG-I [41]. NS1 interferes with the assembly of the IFN-β enhanceosome, thereby limiting IFN-β production [3]. NS1 is able to directly interact with several antiviral factors such as RIG-I and PKR to sequester their antiviral activity [4], [5], [6]. For example, NS1 binds to a linker region in PKR and prevents a conformational change that is normally required for release of PKR auto-inhibition [6]. Because of the importance of NS1 in antagonizing the IFN-mediated antiviral response, we determined whether NS1 interacted with hGBP1 to interfere with the anti-IAV activity of hGBP1. NS1 interacted with hGBP1 as demonstrated by immunoprecipitation and BiFC assay (Fig. 4). The binding of NS1 to hGBP1 resulted in a significant reduction in the GTPase activity (Fig. 7) and antiviral activity of hGBP1 (Fig. 8). These findings indicated that NS1 is involved in inhibition of the hGBP1-mediated antiviral response. This further reinforced the concept that NS1 plays a key role in antagonizing the IFN-mediated antiviral response.

In addition to the requirement of K51 of hGBP1 for inhibiting viral replication (Fig. 3), K51 was required for interaction between hGBP1 and NS1. Mutation of K51 in hGBP1 abolished the interaction between NS1 and hGBP1 (Fig. 4B and 4C; Fig. 6). These results suggested that K51 is a potential target residue for NS1 to antagonize hGBP1-mediated antiviral activity. Because K51 is involved in GTP-binding, dimerization, and GTP hydrolysis [31], NS1 binding might directly or spatially “mask” K51, blocking GTP-binding, dimerization, or GTPase activity, which are essential for hGBP1-mediated antiviral activity. In addition, we found that the hGBP1-binding region was between residues 123 and 144 in the effector domain of NS1 (Fig. 5). This region in NS1 overlaps with a region (residues 123–127) required for PKR-binding [5] suggesting that this region is important for NS1 to antagonize the host antiviral response and is a potential target for the development of new antiviral drugs. Because NS1 is the main antagonist of IFN-mediated antiviral activity, it is a potential target for anti-influenza drug design [3].

Although hGBP1 exhibits antiviral effects, the basis of this effect is unknown [18]. The hGBP1 has an anti-proliferative activity that might help limit the cell-to-cell spread of progeny virus [8]. NS5B protein of HCV interacts directly with hGBP1 and inhibits hGBP1-mediated antiviral activity. This interaction is thought to suppress the biochemical activity of NS5B, a RNA-dependent RNA polymerase of HCV that is important in viral replication [17]. The well-known antiviral protein MxA, a member of a GTPase family, sequesters the nucleocapsids of Thogoto virus (an influenza virus-like orthomyxovirus) from nuclear import, preventing transcription of the viral genome [42]. In this study, a fraction of NS1 was sequestered by hGBP1 in the cytoplasm (Fig. 4C and Fig. S1). NS1 is predominantly localized in the nucleus of IAV-infected cells where it plays various roles in regulating viral replication [3]. The cytoplasmic sequestration of NS1 by hGBP1 could reduce nuclear NS1 and interfere with the regulatory role of NS1 in viral replication.

In conclusion, in response to IAV infection, hGBP1 was significantly upregulated at both the transcriptional and protein levels. Overexpression of hGBP1 inhibited IAV replication and K51 of hGBP1 was essential for inhibition of IAV replication. Viral NS1 interacted with hGBP1. K51 of hGBP1 and the region between residues 123 and 144 in NS1 were identified as essential for the interaction between NS1 and hGBP1. NS1 binding to hGBP1 resulted in a significant reduction in the GTPase activity and anti-IAV activity of hGBP1. These findings indicated that hGBP1 contributed to the host immune response against IAV replication and that hGBP1-mediated antiviral activity was antagonized by NS1 via binding to hGBP1.

## Supporting Information

Figure S1 **Indirect immunofluorescence assay for detecting colocalization of NS1 and hGBP1.** A549 cells were transfected with plasmid Myc-hGBP1-wt or Myc-vector and incubated for 12 h and infected with A/Swine/Jiangsu/2/2006 (H3N2 subtype) at MOI = 1 or mock-infected and incubated for 24 h. Cells were double-immunostained for Myc-hGBP1 (red) and NS1 (green). Nuclei were counterstained with DAPI (blue).
(TIF)

Figure S2 **Schematic illustration of BiFC assay of interaction between hGBP1 and NS1.** Construction of expression plasmids for BiFC assay was performed as previously described [27]. Sequences encoding the N-(amino acids 1–173) and C- (amino acids 174–239) terminal fragments of VFP were fused by a short linker to hGBP1 (hGBP1-VN) and NS1 gene (NS1-VC), respectively. The combination of hGBP1-VN and NS1-VC triggered a strong fluorescence emission.
(PPT)

Table S1 **The sequences of primer used in this study.**
(DOC)
