# Supplementary figures and images for: Nonstructural Protein 1 of Influenza A Virus Interacts with Human Guanylate-Binding Protein 1 to Antagonize Antiviral Activity
Source: PLoS One. 2013 Feb 6;8(2):e55920. doi: 10.1371/journal.pone.0055920 (PMC3566120; doi:10.1371/journal.pone.0055920)

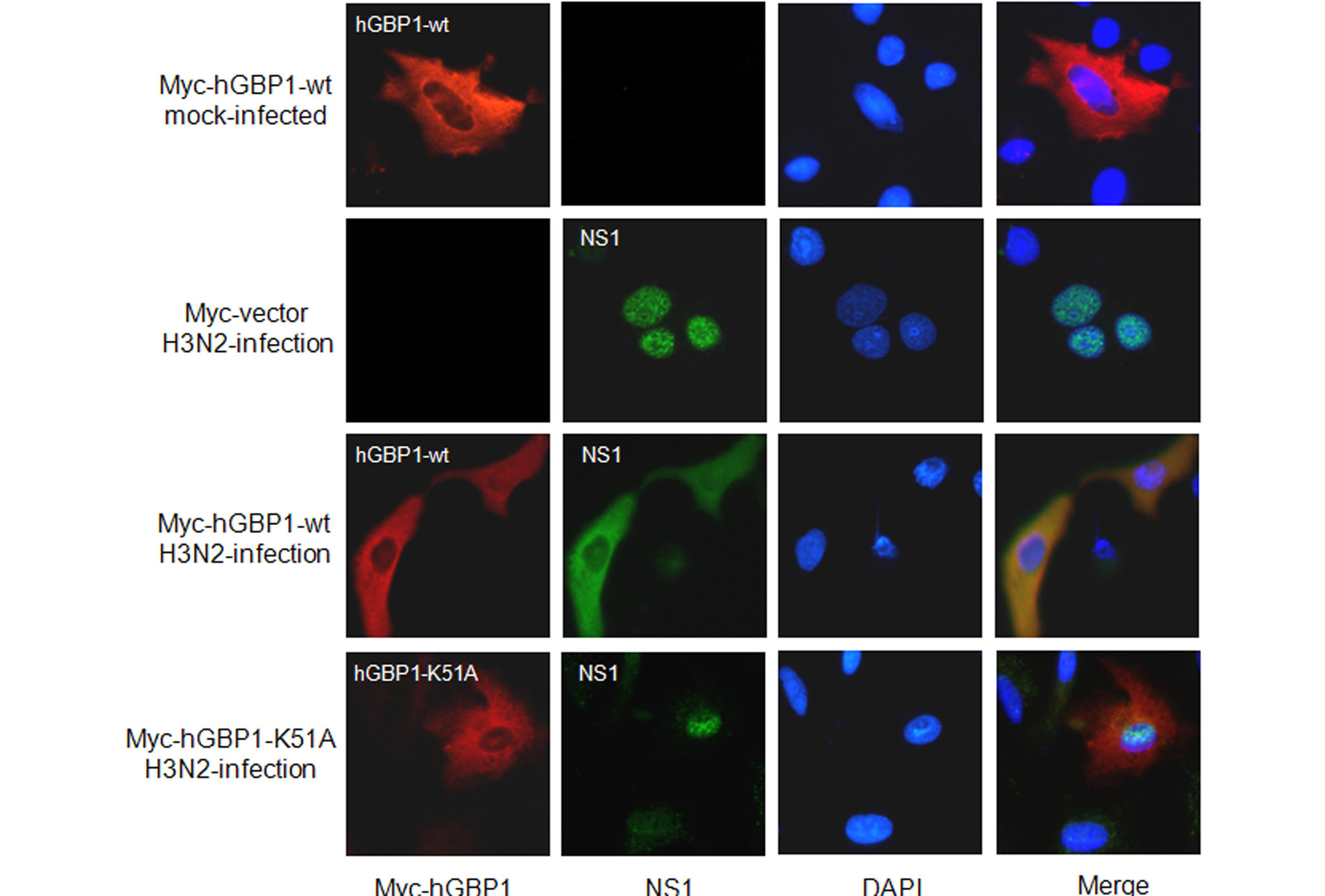

Supplement: Figure S1 — Indirect immunofluorescence assay for detecting colocalization of NS1 and hGBP1. A549 cells were transfected with plasmid Myc-hGBP1-wt or Myc-vector and incubated for 12 h and infected with A/Swine/Jiangsu/2/2006 (H3N2 subtype) at MOI = 1 or mock-infected and incubated for 24 h. Cells were double-immunostained for Myc-hGBP1 (red) and NS1 (green). Nuclei were counterstained with DAPI (blue). (TIF) [file pone.0055920.s001.tif]

## Slide 1
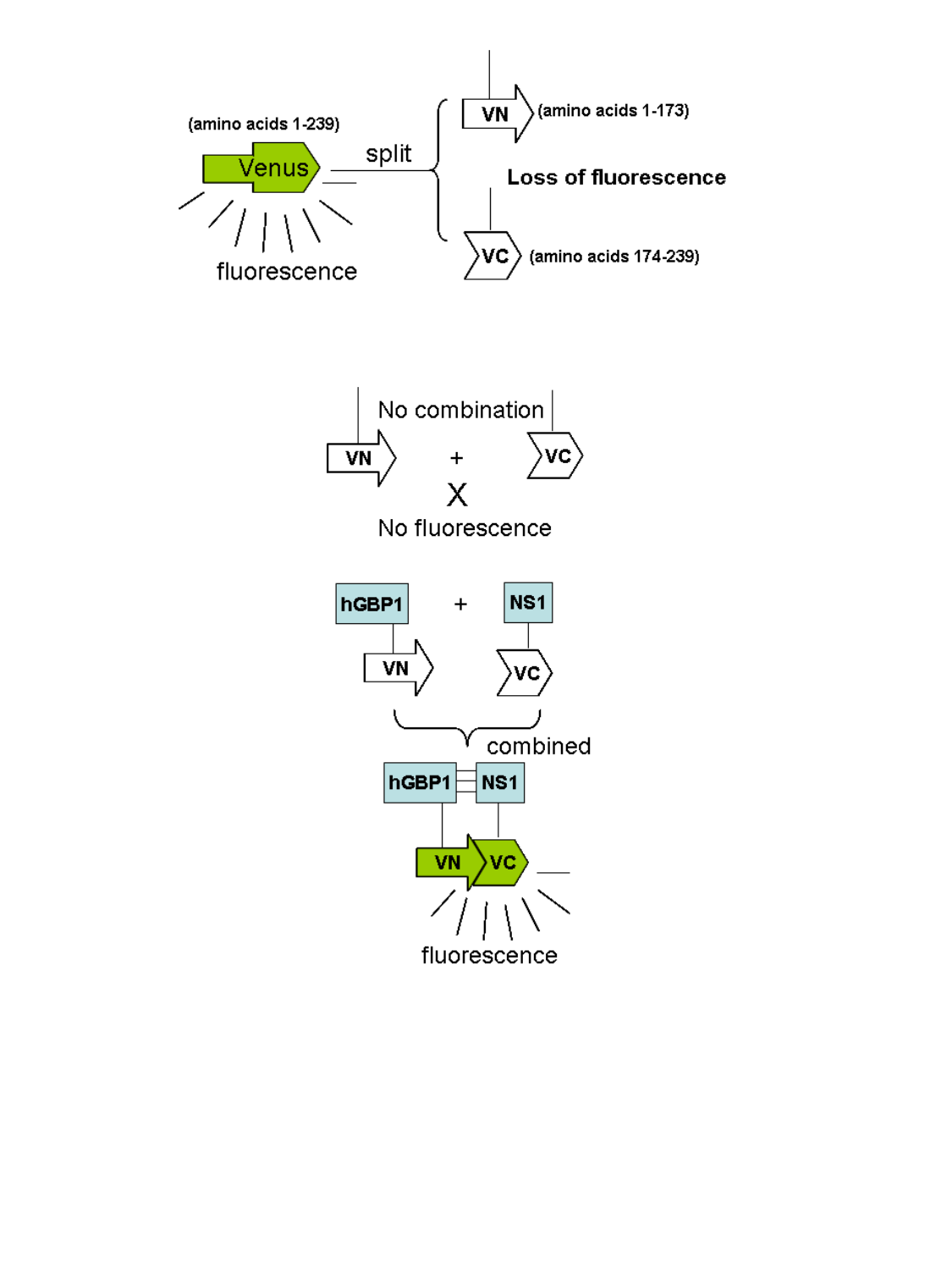

Supplement: Figure S2 — Schematic illustration of BiFC assay of interaction between hGBP1 and NS1. Construction of expression plasmids for BiFC assay was performed as previously described [27]. Sequences encoding the N-(amino acids 1–173) and C- (amino acids 174–239) terminal fragments of VFP were fused by a short linker to hGBP1 (hGBP1-VN) and NS1 gene (NS1-VC), respectively. The combination of hGBP1-VN and NS1-VC triggered a strong fluorescence emission. (PPT) [file pone.0055920.s002.ppt]
