# Supplementary material for: Nonstructural Protein 1 of Influenza A Virus Interacts with Human Guanylate-Binding Protein 1 to Antagonize Antiviral Activity
Source: PLoS One. 2013 Feb 6;8(2):e55920. doi: 10.1371/journal.pone.0055920 (PMC3566120; doi:10.1371/journal.pone.0055920)
Supplement: Table S1 — The sequences of primer used in this study. (DOC) [file pone.0055920.s003.doc]

| Gene name |  | Primer sequence (5’ to 3’) |
| --- | --- | --- |
| hGBP1 | Forward primer | CGAGGGTCTGGGAGATGTAG |
|  | Reverse primer | TAGCCTGCTGGTTGATGGTT |
| HA | Forward primer | TGAACTATTACTGGACCTTGCT |
|  | Reverse primer | CTCCTATTGTGACTGGGTGTAT |
| NP | Forward primer | CTCATCCTTTATGACAAAGAAG |
|  | Reverse primer | AGATCATCATGTGAGTCAGAC |
| GAPDH | Forward primer | CGGGAAGCTTGTGATCAATGG |
|  | Reverse primer | GGCAGTGATGGCATGGACTG |
